# Acquired Factor XI Inhibitor Presenting as Spontaneous Bilateral Subdural Hematoma in an Elderly Patient

**DOI:** 10.1155/2014/626831

**Published:** 2014-11-06

**Authors:** Natale Vazzana, Luca Scarti, Chiara Beltrame, Antonella Picchi, Gianni Taccetti, Alberto Fortini

**Affiliations:** ^1^Department of Internal Medicine, “S. Giovanni di Dio” Hospital, Via di Torregalli, 3-50143 Florence, Italy; ^2^Department of Clinical Chemistry, “S. Giovanni di Dio” Hospital, Via di Torregalli, 3-50143 Florence, Italy

## Abstract

Development of autoantibodies against coagulation factors is an uncommon bleeding disorder associated with cancer, autoimmune conditions, pregnancy, or no apparent disease. Spontaneous FVIII inhibitors are the most frequently encountered; those against FXI have been only anecdotally reported. We report a case of acquired FXI inhibitor presenting as fatal intracranial spontaneous bleeding in an elderly patient with history of cancer and previous transfusions. Few cases of acquired FXI inhibitor have been reported in association with connective tissue disease, cancer, or surgery. Bleeding includes mucocutaneous bleeding, postsurgical hemorrhage, or life-threatening events. Treatment consists of arresting the bleeding and inhibitor eradication. High degree of suspicion is essential to promptly diagnose and treat this uncommon condition.

## 1. Introduction

Immune-mediated development of autoantibodies against coagulation factors in patients without congenital deficiency is a rare but potentially life-threatening bleeding disorder [1]. Acquired hemophilia A (AHA) is the most common form of the disorder and is caused by inhibitory antibodies that neutralize coagulation factor VIII (FVIII) activity [2]. AHA has been associated with malignancy, autoimmune disorders, pregnancy, multiple transfusions, or no apparent disease [3]. Inhibitors against other clotting factors are much rarer [1]; in particular those against FXI have been only anecdotally reported [4–12].

Here we report a case of acquired FXI inhibitors presenting as spontaneous intracranial bleeding in an elderly patient with history of cancer and briefly review current literature on clinical characteristics and management strategies of this uncommon condition.

## 2. Case Presentation

A 90-year-old man presented with decreased level of consciousness and generalised tonic-clonic seizure. He had a history of mild cognitive impairment, myocardial infarction, recurrent syncope, and resected colorectal and bladder cancer two years before, with postsurgical transfusion of six units of packed red blood cells. He did not have hypertension or diabetes and did not smoke. There was no family history of bleeding disorders or altered coagulation tests. His medications included low-dose aspirin, amiodarone, and a statin.

The patient had been in his usual state until 24 hours before this presentation, when worsening confusion, inability to walk, and lethargy developed. There was no recent head trauma. On examination, he was afebrile and unresponsive to deep painful stimuli, with mid-dilated fix pupils and periodic breathing. The arterial blood pressure was 170/100 mmHg, the pulse 60 beats per minute, and the oxygen saturation 97% while he was breathing ambient air. During examination he had a generalized convulsive seizure.

The blood levels of glucose, creatinine, alanine aminotransferase, total bilirubin, sodium, potassium, calcium, and lactic acid were normal. Serum protein electrophoresis showed polyclonal hypergammaglobulinemia without a monoclonal component. The coagulation tests revealed prolonged activated partial thromboplastin time (aPTT: 51 sec, reference range 22–34 sec). Other test results are shown in Table 1.

**Figure 1:**
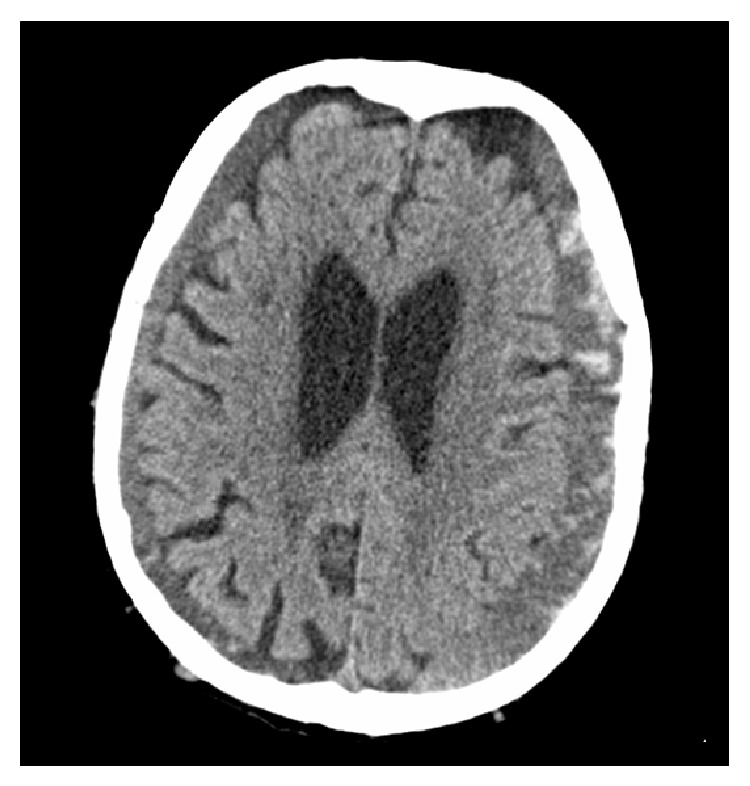
Axial nonenhanced cranial CT scan performed on admission, showing bilateral subdural hematoma with signs of recent bleeding.

Computed tomography of the brain, performed without the administration of contrast material, showed bilateral subdural hematoma with signs of recent bleeding (Figure 1).

Intravenous mannitol was administered and additional blood samples were obtained for further coagulation studies. Despite this treatment, clinical conditions did not improve and the patients died few hours after admission. No hemostatic therapy was administered. Laboratory tests showed (a) prolonged aPTT which could not be corrected by mixing with normal plasma, (b) absence of lupus anticoagulant, and (c) reduced FXI activity (31%, reference range 70–150) due to a low-titer FXI inhibitor (≅1 Bethesda Unit).

## 3. Discussion

Acquired hemophilia should be suspected in presence of unexpected bleeding and a prolonged aPTT [2]. Early recognition, prompt diagnosis, and appropriate treatment are critical to improve the outcomes. Nevertheless, morbidity and mortality are high due to severe bleeding, delayed diagnosis, advanced age, and underlying disorders [2].

Acquired FVIII inhibitor is the most common autoantibody affecting the clotting cascade, with AHA estimated incidence of 1 to 4 per million/year [1]. Guidelines on diagnosis and management of AHA have been recently published [1]. Acquired FIX inhibitors are much rarer, and only few case reports [4–11] and series [11, 12] have been published.

Here we reported a case of acquired inhibitor-related FXI deficiency with fatal intracranial spontaneous bleeding in a patient with advanced age and history of cancer.

FXI inhibitors have been mostly reported in subjects with congenital FXI deficiency after plasma exposure and in presence of specific FXI mutations [13, 14]. Although spontaneous hemorrhages are uncommon in such patients, bleeding after surgery or trauma can be severe [13] and may require specific bypassing treatment [15].

Acquired FXI inhibitors in patients without congenital FXI deficiency have been associated with systemic lupus erythematosus (SLE) [8, 11], hematopoietic malignancies [5, 6, 9], solid cancer [7], inflammatory bowel disease [7], chlorpromazine-treatment [4], and pelvic surgery [10]. Patients usually present with isolated prolonged aPTT not corrected by mixing with normal plasma [1, 2]. In some cases, both prothrombin time and aPTT were prolonged due to multiple coagulation factor inhibitors [4, 8]. As in other autoimmune disorders, inhibitors formation is thought to be related to immune system dysfunction, with aberrant rupture of tolerance to FXI [16]. The association between active or occult cancer and acquired coagulation factor inhibitors is well established [17]. Few cases of transfusion-associated AHA have also been reported [3, 18]. The time between first transfusion and AHA diagnosis ranges from few days to several years [18]. According to these reports, in the present case, the history of multiple transfusions could be also implicated in inhibitor formation.

Bleeding symptoms of acquired FXI deficiency are poorly related to residual FXI activity [1] and include mild or absent bleeding [4, 6, 7], mucocutaneous bleeding [8], postsurgical hemorrhage [5, 10], and life-threatening [11] or fatal intracranial bleeding, as here reported. Unlike AHA, soft tissue and deep muscle bleeding are uncommon in acquired FXI deficiency [2]. Coexistent risk factors such as surgery, trauma, antiplatelet agents, uncontrolled hypertension, or associated hemostatic alterations can modulate the bleeding risk [19]. In the present case, concomitant aspirin treatment and advanced age may explain why bleeding occurred in absence of severe FXI deficiency.

Standard first-line treatment for AHA consists of bypassing agents (activated prothrombin complex concentrates (aPCC) or recombinant activated FVII (rFVIIa)) to control bleeding and steroids with or without cyclophosphamide to eradicate inhibitors [1, 2]. Promising results have also been reported with the use of rituximab [1]. Unlike AHA, there is no consensus on the optimal treatment of acquired FXI inhibitors [1] and most data are derived from treatment of patients with congenital FXI deficiency, with or without secondary inhibitors [19]. Proposed treatment includes (a) antifibrinolytic agents [10, 19], aPCC [20], or rFVIIa [15] for arresting the bleeding, (b) corticosteroids [4, 5, 7–9, 11, 12], azathioprine [6, 8, 11], intravenous immunoglobulins [6, 21], plasma-exchange [21], or rituximab [8] for inhibitor eradication, and (c) specific treatment of the underlying immunologic disorder [5, 8]. In the present report, no hemostatic therapy was administered because of unavailability of coagulation tests, severe disability, and rapid clinical deterioration and death.

A limitation of current report is that family members were not directly tested for factor XI deficiency; in fact, despite the normality of routine coagulation tests, the possibility of an undiagnosed inherited FXI deficiency could not be definitely excluded [15].

In conclusion, acquired factor FXI inhibitors formation is a rare event, which needs to be promptly recognized and managed. There is a polymorphic range of hemorrhagic symptoms and underlining diseases. High degree of suspicion is essential to detect this condition. The optimal hemostatic and eradication therapy should be individualized according to the bleeding severity and the associated disorders.

## Figures and Tables

**Table 1:** Laboratory data.

Variable	18 months before	Admission	Reference range
Hematocrit, %	38.1	28.2	39.0–50.0
Hemoglobin, g/dL	12.4	9.2	13.2–17.0
platelet count, ×10^9^/L	435	200	150–400
PT, %	93	75	70–110
INR	1.06	1.18	
aPTT, sec	25	51	22–34
Fibrinogen, mg/dL	—	710	200–420
FVIII, %	—	263	70–150
FIX, %	—	95	70–150
FXI, %	—	31	70–150
Lupus anticoagulant	—	absent	absent
Total protein, g/dL	5.1	6.3	6.1–8.1
Serum protein electrophoresis			
Albumin, %	—	36.6	55.8–66.1
alpha1, %	—	6.7	2.9–4.9
alpha2, %	—	11.5	7.1–14.8
beta1, %	—	6.6	4.7–7.2
beta2, %	—	7.2	3.2–6.5
gamma, %	—	31.4	11.1–18.8
